# Ballistic Deficit Pulse Processing in Cadmium–Zinc–Telluride Pixel Detectors for High-Flux X-ray Measurements

**DOI:** 10.3390/s22093409

**Published:** 2022-04-29

**Authors:** Antonino Buttacavoli, Fabio Principato, Gaetano Gerardi, Manuele Bettelli, Andrea Zappettini, Paul Seller, Matthew C. Veale, Silvia Zanettini, Leonardo Abbene

**Affiliations:** 1 Department of Physics and Chemistry (DiFC)—Emilio Segrè, University of Palermo, Viale delle Scienze, Edificio 18, 90128 Palermo, Italy; antonino.buttacavoli@unipa.it (A.B.); fabio.principato@unipa.it (F.P.); gaetano.gerardi@unipa.it (G.G.); 2 IMEM/CNR, Parco Area delle Scienze 37/A, 43100 Parma, Italy; manuele.bettelli@imem.cnr.it (M.B.); andrea.zappettini@imem.cnr.it (A.Z.); 3 UKRI Science & Technology Facilities Council, Didcot OX11 0QX, UK; paul.seller@stfc.ac.uk (P.S.); matthew.veale@stfc.ac.uk (M.C.V.); 4 due2lab s.r.l., Via Paolo Borsellino 2, 42019 Scandiano, Italy; zanettini@due2lab.com

**Keywords:** CZT detectors, CdTe detectors, X-ray and gamma ray detectors

## Abstract

High-flux X-ray measurements with high-energy resolution and high throughput require the mitigation of pile-up and dead time effects. The reduction of the time width of the shaped pulses is a key approach, taking into account the distortions from the ballistic deficit, non-linearity, and time instabilities. In this work, we will present the performance of cadmium–zinc–telluride (CdZnTe or CZT) pixel detectors equipped with digital shapers faster than the preamplifier peaking times (*ballistic deficit pulse processing*). The effects on energy resolution, throughput, energy-linearity, time stability, charge sharing, and pile-up are shown. The results highlight the absence of time instabilities and high-energy resolution (<4% FWHM at 122 keV) when ballistic deficit pulse processing (dead time of 90 ns) was used in CZT pixel detectors. These activities are in the framework of an international collaboration on the development of spectroscopic imagers for medical applications (mammography, computed tomography) and non-destructive testing in the food industry.

## 1. Introduction

Photon counting detectors with energy resolving capabilities, typically termed energy resolved photon counting (ERPC) detectors, have been recently developed for high-flux spectroscopic X-ray imaging, with a strong impact in several fields, from diagnostic medicine to synchrotron applications and non-destructive testing (NDT) in the food industry [1,2,3,4,5,6,7,8,9]. Direct semiconductors represent the key detector materials, with particular emphasis on high-Z and wide-bandgap compound semiconductors [10,11,12,13,14] for high-resolution performance near room-temperature conditions. Currently, cadmium telluride (CdTe) and cadmium–zinc–telluride (CdZnTe or CZT) gave the best performances, in terms of crystal growth and device technology [1,2,3,4,5,6,7,8,9,10,11]. In particular, unsurpassed performance, in terms of both detection efficiency (>90%) and energy resolution (<1.5% FWHM at 60 keV), is obtained with thin CdTe pixel detectors (thickness < 1 mm) with Schottky electrical contacts up to 70 keV [15,16,17,18]. However, the use of thicker Schottky CdTe detectors for high energies is limited by the presence of time instabilities due to the well-known bias-induced polarization phenomena [6,19,20,21,22]. At high X-ray energies (>100 keV), interesting energy resolutions (<4% FWHM at 122 keV) [23,24,25] are obtained with thick CdTe/CZT pixel detectors with quasi-ohmic electrical contacts that are immune to the bias-induced polarization effects.

When high-energy resolution and high output counting rates (throughput) are required in high-flux X-ray measurements, pile-up and dead time effects must be mitigated. This can be obtained by using detectors with short charge collection times and short peaking times from both the charge sensitive preamplifiers (CSPs) and the shaping amplifiers. The charge collection time (*T_D_*) of a detector is fixed by the maximum electric field between the electrodes, the leakage current, the charge transport properties of the electrons/holes, and the detector thickness, selected in agreement with the required detection efficiency. The peaking time (*T_CSP_*) of the pulses from the CSPs is slightly greater than the *T_D_*; the result of time integration, due to capacitive effects and gain constraints, often produces a *T_CSP_* greater than *T_D_*. If the energy losses due to the *ballistic deficit* [26,27,28] are to be avoided, the *golden rule* for the shaping amplifiers is to produce pulses with peaking times (*T_S_*) greater than the peaking times *T_CSP_* of the CSP output pulses. Ballistic deficit arises when the peaking times (*T_S_*) of the shaped pulses become comparable with the *T_CSP_*, causing severe energy losses and fluctuations. Therefore, the optimum *T_S_* is always selected greater than the *T_CSP_*, looking for the best trade-off between the energy resolution and dead time/pile-up effects.

Recently, 3-mm thick cadmium telluride (CdTe) detection systems, using digital shapers faster than the peaking times *T_CSP_* of the CSP output pulses (*ballistic deficit pulse processing*), were proposed for high throughput X-ray measurements up to 150 keV [29,30,31]. The systems allow X-ray spectra measurements at high fluxes, with a dead time less than 100 ns and an energy resolution of 8% FWHM at 122 keV. However, the too-fast shaping produces time instabilities in the measured X-ray spectra, on the contrary to what happens when the proper slow shaping is used. These critical issues arise from small changes to the electric field lines with time in quasi-ohmic CdTe detectors [29], producing amplitude variations only on the first part of the leading edge of the CSP output pulses. Because the last part of the leading edge, up to the full amplitude of the pulses, is not influenced by these electric field changes, the time instabilities are not visible when the proper slow shaping is used.

In this scenario, it would be interesting to investigate the presence of any time instabilities in CZT pixel detectors working in the *ballistic deficit regime.*

In this work, we present the performance of several CZT pixel detectors working in the ballistic deficit regime. The effects on energy resolution, throughput, energy-linearity, time-stability, charge sharing, and pile-up are shown. The key results highlight the absence of time instabilities and high-energy resolution when ballistic deficit pulse processing is used in CZT pixel detectors.

## 2. Materials and Methods

### 2.1. The CZT Pixel Detectors

**Figure 1:**
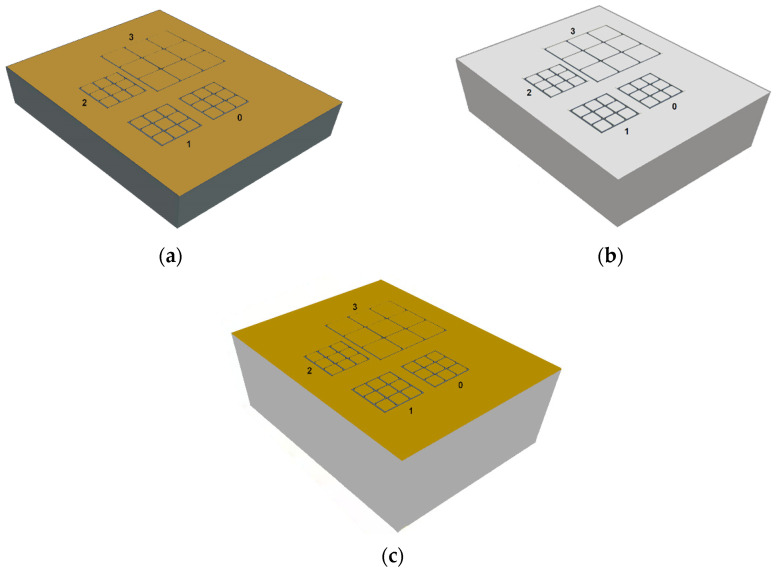
The anode layout of the three CZT pixel detectors. (**a**) The 1-mm thick B-VB CZT pixel detector, (**b**) the 2-mm thick HF-CZT pixel detector, and (**c**) the 3-mm LF-CZT pixel detector. The different colours highlight the related differences in both the CZT crystals and electrical contacts.

The measurements involved CZT pixel detectors with quasi-ohmic electrical contacts (Au, Pt) characterized by different CZT crystals, with thicknesses between 1 and 3 mm. The 1-mm thick CZT detector was based on a boron oxide encapsulated vertical Bridgman (B-VB) CZT crystal, developed at IMEM-CNR of Parma with the collaboration of the due2lab company [32,33,34]. CZT detectors with gold electroless contacts are routinely fabricated at IMEM-CNR and are characterized by low leakage currents at room temperature (<1 nA cm^−2^ at 1000 V cm^−1^) [33,34,35]. In particular, 4% AuCl_3_ methanol solution was used for the cathode electrodes, while the anode patterns were obtained by photolithography and the passivation procedure was performed with an aqueous solution of H_2_O_2_ at 10% for 5 min. The thick detectors were based on travelling-heater-method THM-CZT crystals provided by Redlen Technologies (Saanichton, BC, Canada) [36,37,38,39]. In particular, besides the standard or low flux LF-THM CZT crystals (with enhanced electron charge transport properties), we also used high-flux HF-THM CZT crystals [40,41,42,43], recently fabricated by Redlen and characterized by enhanced hole charge transport properties to minimize high-flux radiation-induced polarization effects [44,45,46]. The details of the detectors (CZT crystals and electrical contacts) are better highlighted in Table 1. As shown in Figure 1, all detectors were characterized by four arrays of 3 × 3 pixels with pixel pitches of 500 µm and 250 µm and an inter-pixel gap of 50 µm; the cathode is a planar electrode covering the full crystal area.

### 2.2. The Preamplifiers and the Digital Pulse Processing Electronics

**Figure 2:**
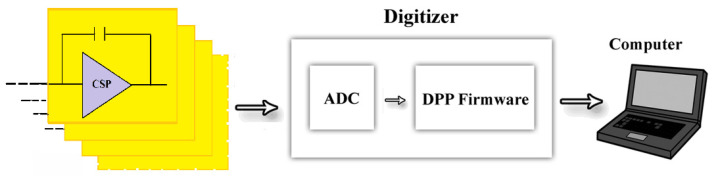
Schematic view of the readout electronic circuit architecture. On the left, there is the charge-sensitive preamplifiers of the PIXIE ASIC [47], where the CZT pixels were flip-chip bonded; the pulse shaping and the pulse height analysis was performed by custom digital pulse processing electronics [48,49,50] controlled through a PC.

Figure 2 shows a schematic view of the readout electronics used for all CZT pixel detectors.

**Figure 3:**
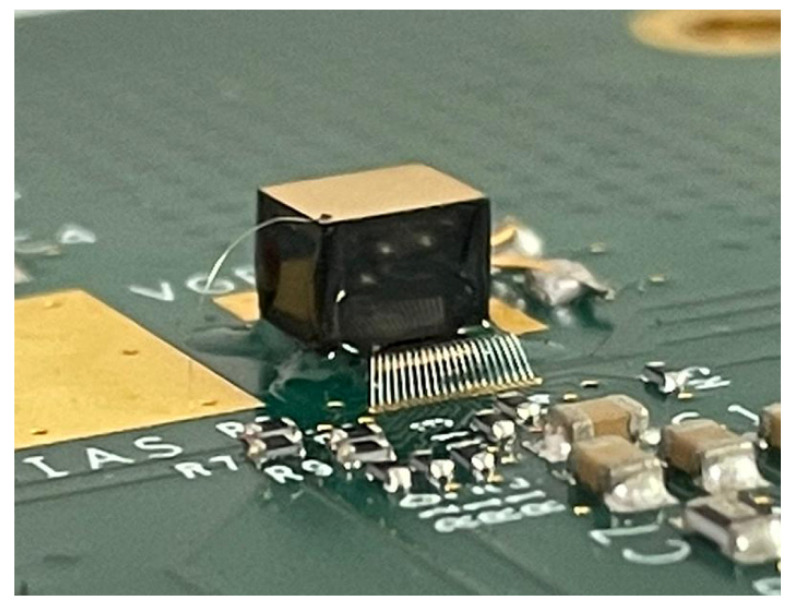
The 3-mm LF-CZT pixel detector flip-chip bonded on the PIXIE ASIC. A bias voltage of −1800 V was supplied by a gold wire glued on the planar cathode electrode, clearly visible in the picture.

The front-end electronics was represented by charge-sensitive preamplifiers (CSPs) without pulse shaping. They were based on a low-noise application specific integrated circuit (PIXIE ASIC) developed at the Rutherford Appleton Laboratory RAL (Didcot, UK) [47]. The PIXIE ASIC was equipped with 36 CSPs arranged in four arrays of 3 × 3 pads, flip-chip bonded directly to the pixels of the detectors (Figure 3).

Each array can be selected by the user providing, simultaneously, nine preamplifier outputs, with no pulse shaping processing. The electronic noise is very low, i.e., with an equivalent noise charge (ENC) less than 80 electrons. In this work, we only used the pixels of the 500-μm arrays.

The pulse shaping processing of the CSP output pulses was performed through a digital approach, using 16-channel digital electronics, developed at DiFC of the University of Palermo (Palermo, Italy) [48,49,50]. The digital electronics were based on commercial digitizers (DT5724, 16-bit, 100 MS/s, CAEN SpA, Italy; https://www.caen.it, accessed on 26 April 2022), where a dedicated firmware was uploaded [48,49,50]. The potentialities of the digital pulse processing approach are now widely recognized [51,52,53]; in our case, the flexibility of this approach, due to the possibility of using different pulse shaping features, was a key point for our investigation.

For each CSP output channel, the digital system performed on-line pulse detection, time-tag triggering, and pulse height analysis. The details of the pulse shaping operations and the outputs from each CSP output channel are described below:

(i) Pulse detection and arrival time estimation; the CSP output waveforms were shaped using the classical single delay line (SDL) shaping technique [26], acting as the classical differentiation; the trigger time was generated and time-stamped through the ARC (amplitude and rise time compensation) timing marker (at the leading edge of the SDL pulses), able to reduce the distortions from time jitters and amplitude and rise time walks [26];
(ii) Pulse height analysis (energy estimation); the detected CSP output pulses with the related arrival times were shaped with a classical trapezoidal filter [26]. We used trapezoidal-shaped pulses with peaking times (*T_S_*) ranging from 30 ns to 1000 ns.

Further details of the digital electronics are reported in our previous works [48,49,50].

### 2.3. Experimental Procedures

**Figure 4:**
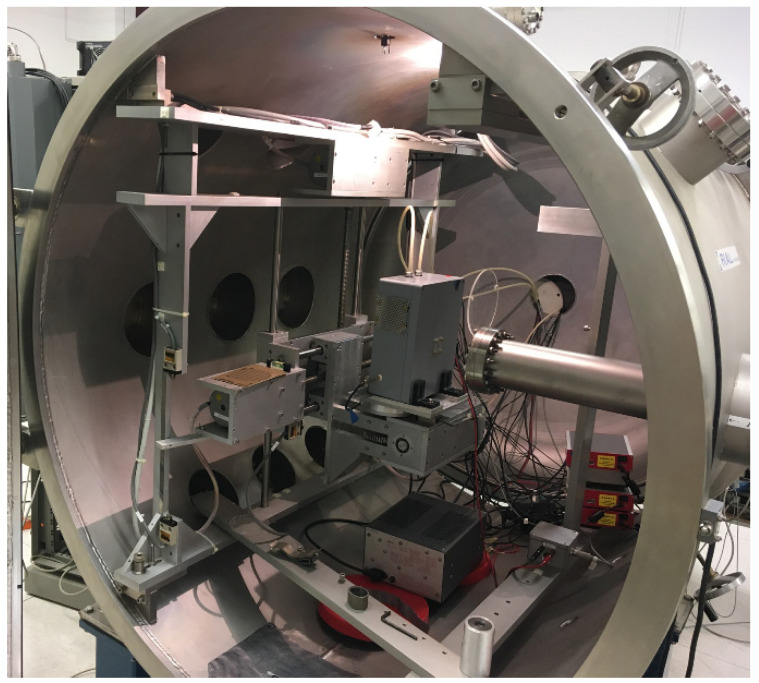
An overview of the experimental setup used for high-flux X-ray measurements at the Livio Scarsi X-ray facility. The CZT pixel detectors (enclosed in the grey rectangular box, together with the preamplifier PIXIE ASIC) were irradiated with Mo target X-rays (the tube on the right side). The red boxes, on the bottom right side, are the digitizers of the digital pulse processing electronics. The detector box was mounted on a micro-translator system, which can be moved in *x*, *y*, and *z* directions with a precision of 10 μm.

The measurements were performed at the laboratory of ionizing radiation detectors of the University of Palermo (Italy). All detectors were irradiated through the cathode electrode with uncollimated radiation sources (^109^Cd, ^241^Am and ^57^Co sources). High-flux measurements with X-ray tubes were conducted at the Livio Scarsi X-ray facility of the University of Palermo [54]. We used X-rays (main fluorescent lines at 17.5 and 19.6 keV) from a Mo target typical of mammographic X-ray beams. An overview of the experimental setup used is shown in Figure 4. All measurements were performed at room temperature (T = 20 °C).

## 3. Ballistic Deficit Pulse Processing in CZT Pixel Detectors: Measurements and Results

In this section, we will show the effects of the ballistic deficit pulse processing approach on the energy resolution, throughput, energy-linearity, time-stability, charge sharing, and pile-up in the CZT pixel detectors. A comparison with the results obtained from an energy resolution pulse processing, optimized for the best energy resolution, will be also presented.

### 3.1. Energy Resolution and Throughput

**Figure 5:**
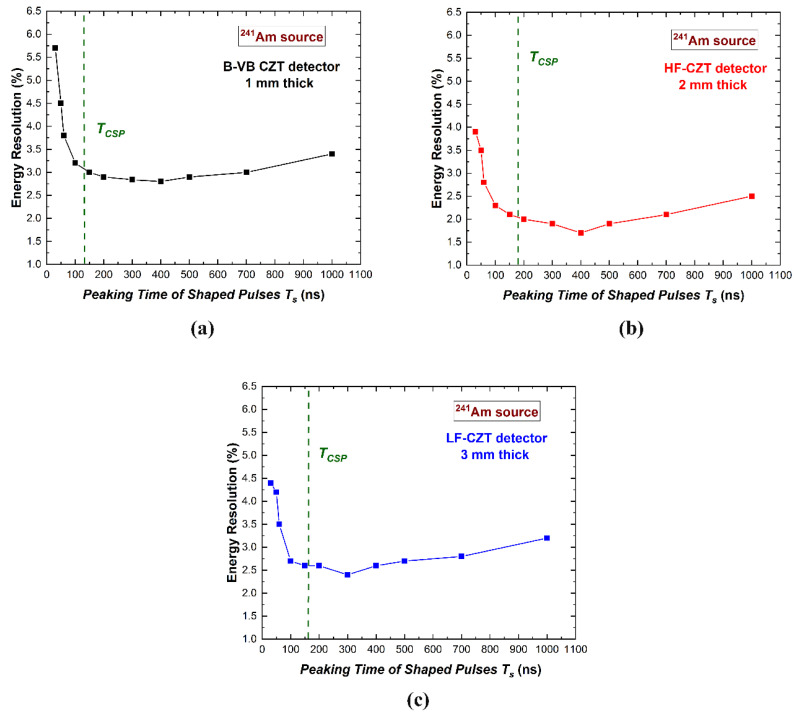
Measurement of the energy resolution (FWHM) at 59.5 keV vs. the peaking time of the shaped pulses. The results for the (**a**) B-VB CZT pixel detector, (**b**) HF-CZT pixel detector, and (**c**) LF-CZT pixel detector. The green dashed vertical lines represent the mean value of the peaking times *T_CSP_* of the CSP output pulses.

The selection of the optimum shaping peaking time value (*T_S_*) is a key procedure for all radiation detectors [26]. Typically, the optimum *T_S_* value is chosen looking for the best energy resolution (energy resolution pulse processing). Figure 5 shows the energy resolution (FWHM) values at 59.5 keV measured at different *T_S_* values. All detectors were characterized by optimum *T_S_* values ranging from 300 ns to 400 ns; these values were always greater than the CSP peaking time *T_CSP_* (green dashed vertical lines), due to the minimization of the ballistic deficit fluctuations. As it is well-known in the literature [55], the optimum *T_S_* values also follow the equilibrium between two main electronic noise components. At high *T_S_* values, the parallel white noise (shot noise mainly due to the leakage current of the detectors) dominated the energy resolution, while at low *T_S_* values, the series white noise (thermal noise due to the drain current of preamplifier input FETs) was the main contributor. In our measurements, the degradation of the energy resolution at *T_S_* < *T_CSP_* was due to both the ballistic deficit and series noise effects.

**Figure 6:**
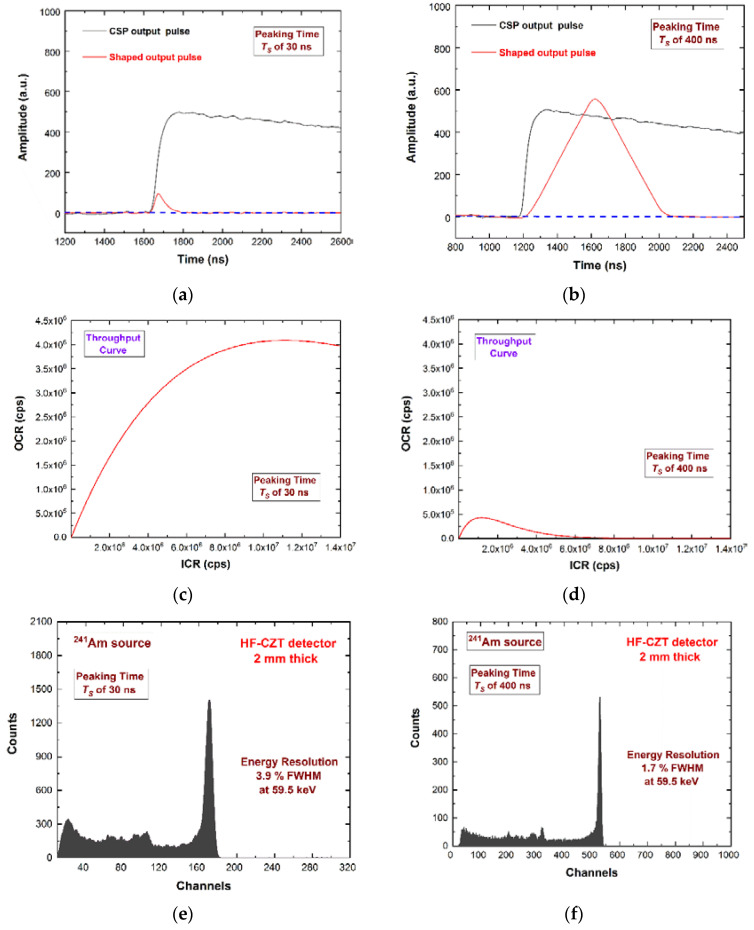
The preamplifier output pulses (black lines) and the shaped output pulses (red lines) with peaking times *T_S_* of (**a**) 30 ns (*ballistic deficit pulse processing*) and (**b**) 400 ns (*energy resolution pulse processing*). The calculated throughput curves, i.e., the output counting rate (OCR) vs. the input counting rate (ICR), with *T_S_* of (**c**) 30 ns and (**d**) 400 ns. The throughput curves were calculated considering paralyzable dead times (the full-time width of the shaped pulses over the threshold) of 90 ns and 850 ns for *T_S_* = 30 ns and 400 ns, respectively. The measured ^241^Am energy spectra with *T_S_* equal to (**e**) 30 ns and (**f**) 400 ns. To optimize the binning of the pulse heights of the shaped pulses at *T_S_* of 30 ns, we used an amplitude gain equal to 2.

In order to increase the throughput of the system (i.e., the ratio between the output and the input counting rate), the *T_S_* should be chosen as low as possible, taking into account the degradation of the energy resolution. In Figure 6, we present the performance of the 2-mm HF-CZT pixel detector, in terms of throughput and energy resolution (^241^Am source).

**Figure 7:**
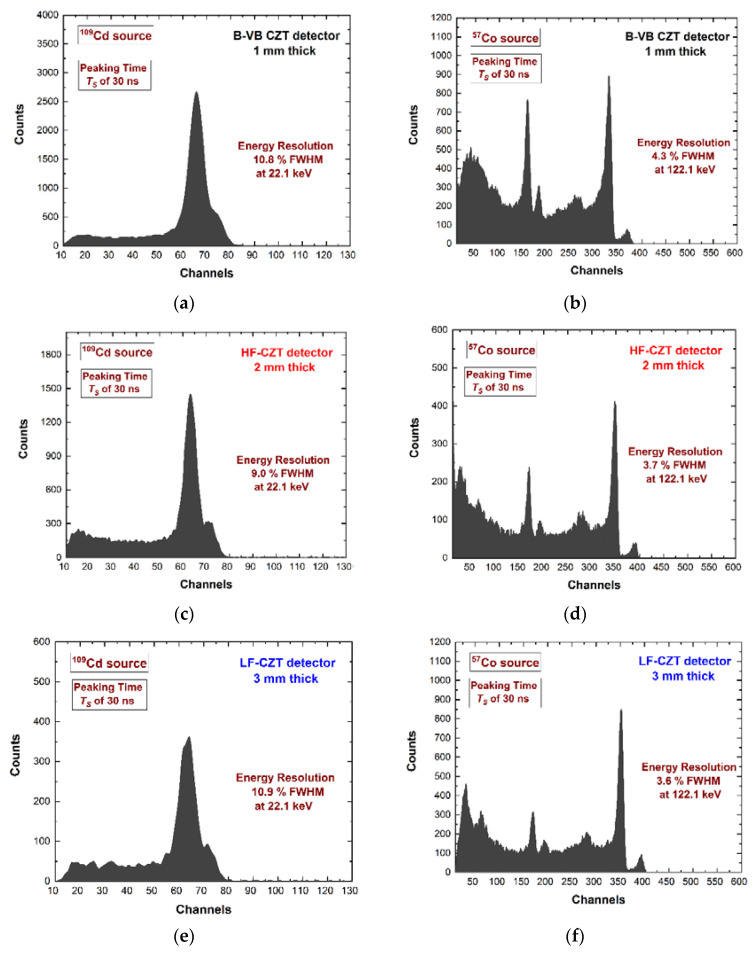
Measured (**a**,**c**,**e**) ^109^Cd and (**b**,**d**,**f**) ^57^Co energy spectra for the three CZT pixel detectors. All spectra were obtained using the ballistic deficit pulse processing with a peaking time *T_S_* of 30 ns (dead time of 90 ns).

We used two different pulse shaping set-ups: the first with a *T_S_* of 30 ns selected for high throughput (ballistic deficit pulse processing), the second with a *T_S_* of 400 ns for the best energy resolution (energy resolution pulse processing). The mean value of the time widths of the shaped pulses over the detection threshold was 90 ns and 850 ns for *T_S_* of 30 ns and 400 ns, respectively. This time width was a dead time for the system and can be modelled as paralyzable dead time [26,56]. If a second pulse arrives while the first pulse is still above the detection threshold, the second pulse overlays the first, and extends the dead time by its width from its arrival time. Because the system counts threshold crossings, it will count only the first pulse. In agreement with the paralyzable dead time model, we calculated the throughput curves at the two-pulse processing set-up. The ballistic deficit pulse processing (dead time of 90 ns) ensured a maximum output counting rate (OCR) of 4.1 Mcps (Figure 6c), while the energy resolution pulse processing was of 0.43 Mcps (Figure 6d). Further energy spectra using the ballistic deficit pulse processing are shown in Figure 7. 

Excellent energy resolution of 3.6% FWHM at 122 keV was obtained with the 3-mm CZT pixel detector. This was an interesting result when compared with the energy resolution of about 8% at 122 keV for the 3-mm CdTe pixel detectors using similar ballistic deficit pulse processing [29].

### 3.2. Energy Linearity and Time Stability

**Figure 8:**
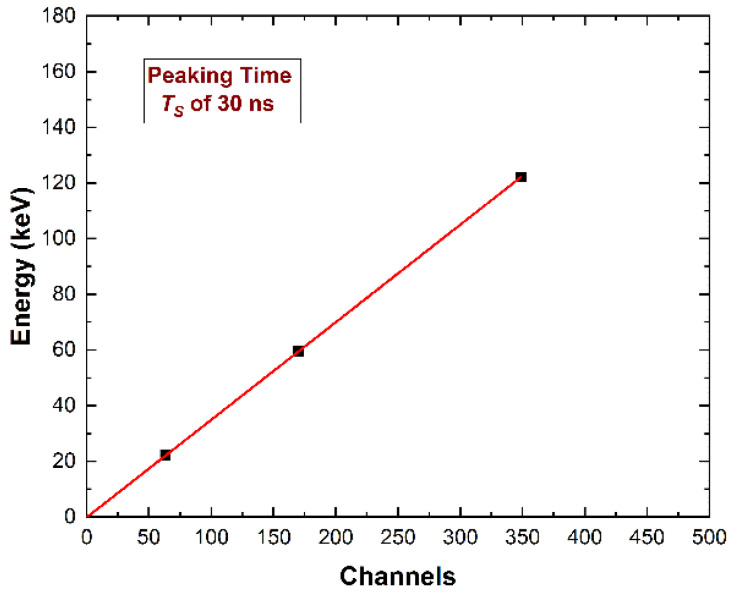
The photon energy vs. the pulse height (channels) obtained using the ballistic deficit pulse processing approach. The linearity with energy was well verified.

The linearity of the ballistic deficit pulse processing with energy was investigated. In particular, the linearity of the pulse heights with the photon energy was experimentally verified for all detectors, as shown in Figure 8.

**Figure 9:**
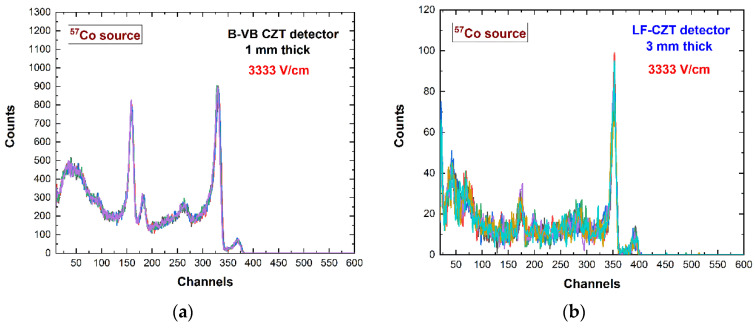
The energy spectra measured with the (**a**) B-VB CZT pixel detector and (**b**) LF-CZT pixel detector within a time window of one hour. For each time window of one hour, we measured six energy spectra with an acquisition time of ten minutes. ^57^Co sources with different activity were used. The time stability was verified.

As discussed in the introduction, CdTe detectors with quasi-ohmic electrical contacts, when working in ballistic deficit regimes, suffer from time instabilities due to the temporal changes of the electric field lines [29]. This was investigated in our detectors working at the same electric field set-up (3333 V/cm) used in CdTe detectors. In particular, Figure 9 shows the ^57^Co energy spectra measured within a time window of one hour. The time stability was well verified, demonstrating the more stable electrical contacts (Au, Pt) and electric field in quasi-ohmic CZT pixel detectors. This also highlighted the absence of temporal changes of the space charge in CZT materials.

### 3.3. Charge Sharing

**Figure 10:**
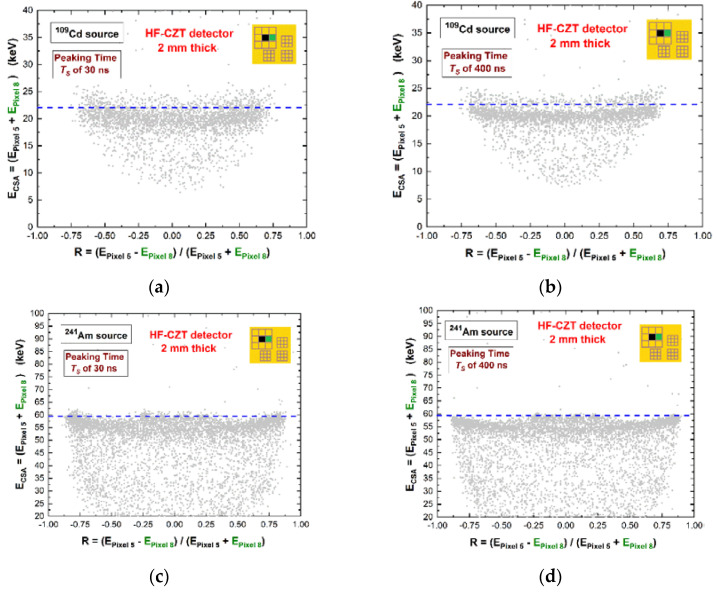
Two-dimensional (2D) scatter plot of the summed energy of the coincidence events (multiplicity m = 2) between pixel no. 5 and pixel no. 8 after, the charge sharing addition (CSA), versus the sharing ratio *R*. (**a**,**c**) Ballistic deficit pulse processing; (**b**,**d**) energy resolution pulse processing. The blue dashed lines represent the true energy.

The effects of the ballistic deficit pulse processing on the charge sharing measurements were also investigated. Typically, charge sharing is strongly present in sub-millimetre CZT pixel detectors. The percentage of charge sharing events is very high, of about 50–60% for the pixels with 500 μm pitches [23,48]. As it is well known, the energy of the shared events can be recovered by summing their energies, measured in temporal coincidence. However, the energy recovered after charge sharing addition (CSA) is often characterized by deficits, due to the presence of charge losses at the inter-pixel gap [57,58,59,60,61,62,63,64,65]. In our case, we investigated the effects of ballistic deficit pulse processing on these charge losses. Figure 10 shows the scatter plots of the summed energy E_CSA_ versus the sharing ratio *R*. The energy E_CSA_ = (E_Pixel5_ + E_Pixel8_) was the sum of the energies of two coincidence events between two adjacent pixels, i.e., after CSA, while the charge sharing ratio *R* was calculated from the ratio between the energy of the two coincidence events, as follows: *R* = (E_Pixel5_ − E_Pixel8_)/(E_Pixel5_ + E_Pixel5_). *R* is typically used to provide information about the interaction position of the shared events within the inter-pixel gap.

**Figure 11:**
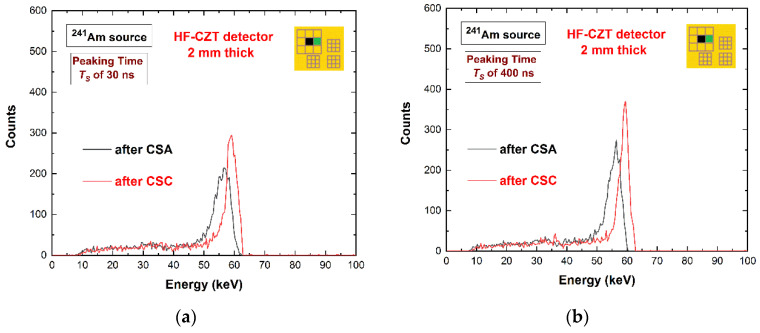
The energy spectra after CSA (black line) and after CSC (red line). (**a**) Ballistic deficit pulse processing; (**b**) energy resolution pulse processing.

The plots on the left side were obtained using ballistic deficit pulse processing; on the right side, we used energy resolution pulse processing. Despite the poor energy distribution of the shared events (poor energy resolution), the ballistic deficit pulse processing did not increase the charge losses after CSA. This result is also shown in Figure 11, where the energy spectra after CSA (black lines) are presented. The charge losses (3 keV at 59.5 keV) were the same for both shaping processing approaches, even if the energy resolution of the energy spectra after CSA was poorer for the ballistic deficit pulse processing. Moreover, the correction of these charge losses was successfully performed for both processing approaches (red lines) through the application of a charge sharing correction (CSC) technique developed by our group and presented in our previous works [23,63]. The best energy resolution of the corrected energy spectra after CSC (red lines) was obtained with energy resolution pulse processing.

### 3.4. High Flux Performance

**Figure 12:**
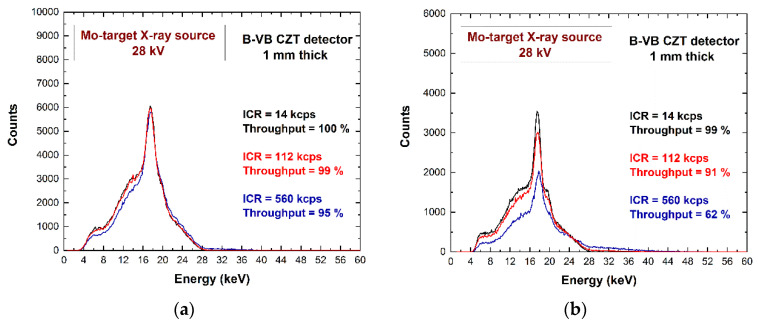
The Mo-target X-ray spectra at different input counting rates (ICRs). (**a**) Ballistic deficit pulse processing; (**b**) energy resolution pulse processing.

The goal of using ballistic deficit pulse processing was to obtain high throughput energy spectra with low pile-up effects and an energy resolution as high as possible. To investigate on the potentialities of this approach at high fluxes, we measured Mo-target X-ray spectra (with fluorescent X-ray lines at 17.5 keV and 19.6 keV) at different rates, up to a maximum saturation rate of the CSPs (600 kcps). The energy spectra, measured using both ballistic deficit and energy resolution pulse processing, are shown in Figure 12. At high rates (560 kcps), the ballistic deficit pulse processing gave the best results: similar energy resolution with the energy resolution pulse processing, high throughput (95%), and low pile-up effects in the spectra. 

On the contrary, the effects of pile-up are visible when the energy resolution pulse processing was used, with the presence of more background events beyond the 28-keV end-point energy of the spectrum (blue line of Figure 12b).

## 4. Discussion

The measurements using the ballistic deficit pulse processing (shaped pulses with time widths of 90 ns) in CZT pixel detectors highlighted the following key results:

- All detectors showed good energy resolutions of about 4% FWHM at 122 keV, in particular when compared with the energy resolution of about 8% obtained with 3-mm CdTe pixel detectors using similar ballistic deficit pulse processing [29]; this is due to the high bias voltage operation of the detectors (>5000 V/cm) which minimizes the changes of the charge collection times and, therefore, the effects of ballistic deficit; moreover, the series noise, very important in this shaping set-up, was also mitigated by the low noise front-end electronics (PIXIE ASICs).
- The linearity of the pulse heights with the energy was verified in all CZT detectors.
- We observed the absence of time instabilities, typically present in CdTe pixel detectors with quasi-ohmic electrical contacts [29]; this demonstrated the time stability of the electric field lines in the quasi-ohmic (Au, Pt) CZT detectors, highlighting the absence of space charge changes with time in CZT materials.
- The ballistic deficit pulse processing did not increase the charge losses after the charge sharing addition (CSA) and the energy recovery was successfully applied.
- At high rates (560 kcps), we measured energy spectra with very high throughput (95%), low pile-up effects, and a similar energy resolution obtained with the energy resolution pulse processing approach; potentially, the dead time of 90 ns, modelled as paralyzable dead time, can ensure a maximum output counting rate (OCR) of 4.1 Mcps.

## 5. Conclusions

In this work, we presented the potentialities of a ballistic deficit pulse processing approach for high-flux X-ray measurements with CZT pixel detectors. This approach consists of using shaped pulses with peaking times less than the peaking times of the preamplifier pulses. Despite the long peaking times of the preamplifiers (150–170 ns), we used digital shaped pulses with peaking times of 30 ns and a dead time less than 90 ns. Interesting energy resolution (4% at 122 keV) was obtained in various CZT pixel detectors, characterized by different thicknesses, crystals, and electrical contacts. The time instabilities, typically present in CdTe detectors, were not observed, demonstrating the absence of space charge changes with time in CZT materials.

Future activities will be focused on the use of new digitizers with a higher sampling frequency (>100 MHz) for performance enhancements in ballistic deficit pulse processing. We believe that the high sampling frequency can improve the pulse height analysis and the energy resolution of the detection systems.

## Figures and Tables

**Table 1:** The key characteristics of the CZT pixel detectors.

CZT Crystals	Mobility-Lifetime Products *μτ* (cm^2^/V)	Electrical Contacts
B-VB CZT (4.25 × 3.25 × 1 mm^3^) IMEM-CNR Parma (Parma, Italy) ^1^ due2lab s.r.l. (Scandiano, Italy) ^1^	*μ_e_τ_e_* 0.6–0.7 × 10^−3^ *μ_h_τ_h_* not measured	gold (Au) electroless quasi-ohmic IMEM-CNR Parma (Italy) ^1^ due2lab s.r.l. (Italy) ^1^
HF-CZT (4.25 × 3.25 × 2 mm^3^) Redlen Technologies (Canada) ^1^	*μ_e_τ_e_* 2–3 × 10^−3^ *μ_h_τ_h_* 1–2 × 10^−4^	platinum (Pt) quasi-ohmic Redlen Technologies (Canada) ^1^
LF-CZT (4.25 × 3.25 × 3 mm^3^) Redlen Technologies (Canada) ^1^	*μ_e_τ_e_* 1–3 × 10^−2^ *μ_h_τ_h_* 2–3 × 10^−5^	gold (Au) electroless quasi-ohmic IMEM-CNR Parma (Italy) ^1^ due2lab s.r.l. (Italy) ^1^

^1^ The manufacturers.
